# Structure and morphology of cellulose fibers in garlic skin

**DOI:** 10.1038/s41598-020-59479-1

**Published:** 2020-02-14

**Authors:** Maria Raimo

**Affiliations:** Consiglio Nazionale delle Ricerche, Istituto per i Polimeri, Compositi e Biomateriali, Via Campi Flegrei, 34-80078 Pozzuoli, NA Italy

**Keywords:** Plant development, Biomaterials

## Abstract

The knowledge of the texture and morphology of cellulose is essential for reliable modelling of cell growth and mechanical resistance of vegetal systems. Microscopic observations on thin layers of the skin of *Allium sativum* have shown elongated structures (i.e. cellulose fibers) imbedded in a matrix of more or less rounded cells. Examination by an optical polarizing microscope (OPM) has shown an intermittent high and low birefringence along fibers. Transversal regions with a reduced brightness along fibers are expected to contain a higher amount of amorphous lignin, hemicelluloses and waxes, some of which might also be birefringent, but at a much lower degree than cellulose. Scanning electron microscopy (SEM) has also evidenced an alternating growth of the fibers. Moreover, the negative sign of birefringence suggests a parallel orientation of cellulose nanofibrils transversally to the fiber axis. The characteristic modulation of intensity along lignocellulosic fibers can be due to variation of the cellulose concentration or orientation, perhaps caused by circadian cycles of temperature and light during growth. Indeed, imperfect orthogonal light can be totally reflected at the interface between regions with different values of the refractive index, contributing to the optical effect of banding.

## Introduction

**Figure 1:**
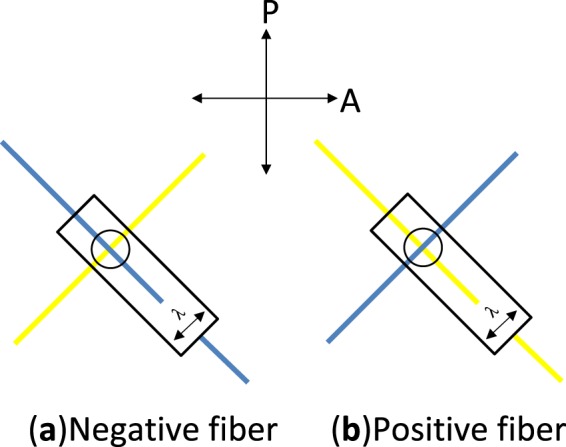
Determination of the birefringence sign of a fiber by means of a full-wave plate. The crossed arrows correspond to the polarizer (P) and analyzer (A), whereas the arrow on the λ plate indicates its slow direction. When the longer side of the frame of the λ plate is parallel to a fiber in the NW-SE direction, the sign is negative if the fiber is blue (**a**), positive is the fiber is yellow (**b**). By rotating the fiber of 90°, the two interference colours are exchanged.

The species of *Allium* have attracted many attentions because of their potential health benefits. The intense analysis of cloves aimed at the identification and characterization of new substances and drugs, whereas stalk and skin have been extensively studied as renewable and non-conventional sources of cellulosic fibers^1,2^. However, at the best of knowledge of the present author, no microscopic investigation on native cellulosic structures of garlic has been performed. The lack of inquiries on non-textile cellulose fibers limits not only information on their growth and morphology, but also on the arrangement of cellulose nanofibrils within them. Since the structure of the cell walls affects considerably the mechanical properties of plant tissues, the knowledge of the orientation of fibrils and the interactions of cellulose with other components allows the definition of a reliable micromechanical model, especially when fibers are unsuitable to be submitted to standard tests^3^. An easily observable optical property of most of solids and, in general, of asymmetric structures formed by oriented rod elements in the liquid state, is birefringence^4,5^. Light emerging from a birefringent substance will not have, in general, a polarization state equal to that of the incident light and, therefore, it will be transmitted through a second orthogonal polarizer. The method used to determine the sign of birefringence of a fiber by means of a full-wave plate, is based on the change from an interference colour to a different colour (the one being a tint of a little lower and the other a tint of a little higher than the interference colour of the sole λ plate^6^), for 90° rotations of the fiber between crossed polarizers^6–8^. Slightly birefringent substances show interference colours, varying with the thickness and the orientation of the optical axis, from dark grey to whitish of the first order, according to Michel-Levy table for mineral identification^8^. Fibers of such substances, when observed between crossed polarizers (N-S direction for the polarizer and W-E direction for the analyzer) with a λ plate placed at 45° (NW-SE direction) according to Fig. 1, turn from blue to yellow and *vice versa*, through magenta, for 90° rotations of the fiber^8–10^.

Previous thermal, electron microscopy and spectroscopic analyses of cellulose fibers of garlic stalk and skin have been made after steam explosion or alkali and acid treatments, in order to separate the different components. Several authors also estimated the composition of garlic stalk and skin. For instance, Reddy and Rhim^1^ found 41.7 ± 2.1% of cellulose, 20.8 ± 1.6% of hemicelluloses,34.5 ± 2.4% of lignin, and 3.0 ± 0.3% of extractives (organics, waxes, pectin, and other impurities) in garlic skin, and similar values have been found by Moreno *et al*.^2^, that also reported a very little variation with the geographic provenience of the crops. Cellulose and hemicellulose are therefore the main potentially birefringent components of garlic skin^11^, respectively as imbedded fibers and hosting matrix, whereas proteins, waxes and other crystalline substances are contained in too low amounts to show birefringence. Most of hemicellulose are, however, amorphous and, hence, cannot contribute to birefringence. On the other hand, cutin and suberin (found in in some plants and bulbs where they act as water and microorganisms barrier^12^) show a lamellar morphology and, if amorphous as mostly considered, they can show birefringence only because of the associated waxes, which in garlic skin are also in too negligible amounts to effectively contribute to birefringence. Transmission electron microscopy (TEM) of suberized layers in cell walls has shown alternant brightness (due to electron-translucence) and darkness (due to electron-opaqueness)^13^. Some authors have attributed bright bands to waxes and dark bands to phenolic substances, whereas recent studies have assumed that regularly packed poly(acylglycerol) segments in the core of suberin form the bright lamellae and the surrounding polyaromatic blocks form the dark lamellae^13^. On the basis of this assumption, suberin should have an ordered structure and, therefore, could show birefringence. However, each lamella observable by TEM has a thickness of only a few nm, whereas the whole poly-lamellar layer has a sub-micrometer width and, therefore, even multi-layered suberin would result too thin to be observable between crossed polarized under an optical microscope. Indeed, suberin is considered to be covalently bounded, through the lignin-like phenolic blocks, to wall cell carbohydrates, thus forming a well dispersed sub-micrometric phase^14^ that cannot show appreciable birefringence^6^. High magnification of garlic skin are not achievable even by SEM because of the thermal instability of the skin. However, since SEM and TEM analysis of vegetal tissues much more resistant than garlic skin show that the typical dimensions of suberin lamellae are of the order of nanometer, even if suberin was present in garlic skin, its lamellar structure could not be observed at the low magnification and resolution offered by an optical microscope. As a consequence, extended optically banded regions with modulate brightness found by OPM in garlic skin are unlikely due to cell walls suberization or to the occurrence of suberin gradients along cellulose fibers. Here peeled skin of garlic has been observed, under an optical polarizing microscope in transmission modality, without any preliminary treatment, in order to preserve the original texture and strength of fibers, and to avoid birefringence artifacts due to mechanical stress^9,15^ or chemical treatments during further handling. Optical properties, namely birefringence patterns, can reveal several details on growth of tissues and crystals^16,17^ and, particularly, provide information on the array of nanofibrils of cellulose within microfibers^18^. These details can be advantageously used to improve models for the prediction of mechanical properties that cannot be trustworthy measured, as those of fragile biological systems.

## Results

### OPM and SEM analysis

**Figure 2:**
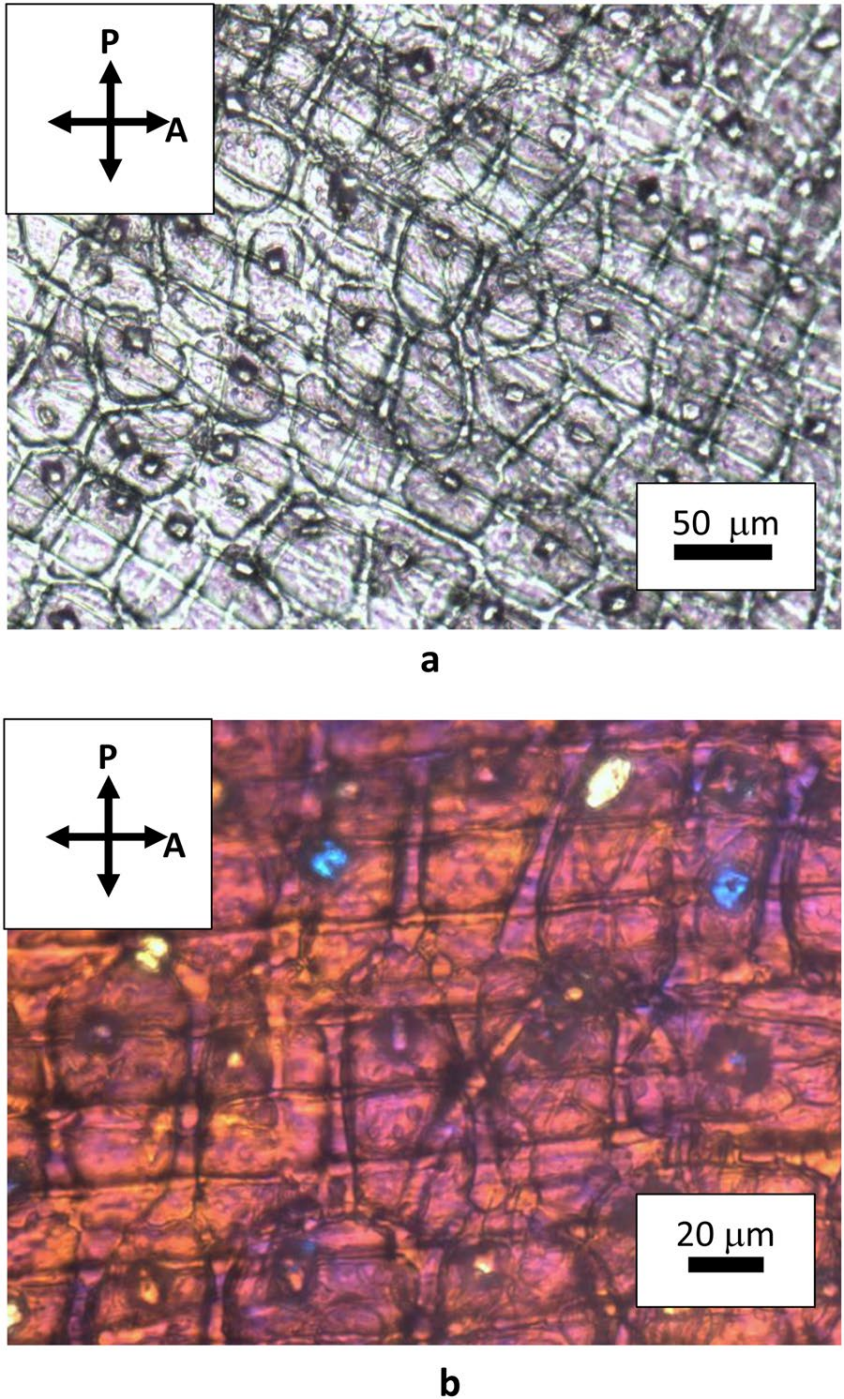
Crossed polarizers micrographs of garlic skin taken (**a**) with an Achroplan 20X/0.40 objective, scale length 50 μm and (**b**) with an Achroplan 40X/0.65 objective in the presence of a λ plate oriented in the NW-SE direction according to Fig. 1, scale length 20 μm.

**Figure 3:**
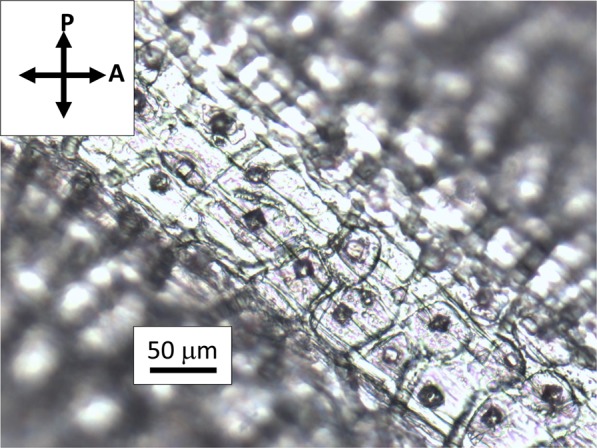
Micrograph showing an elongate structure embedded in a matrix of rounded cells. Objective 20X/0.40, scale length 50 μm, crossed polarizers.

**Figure 4:**
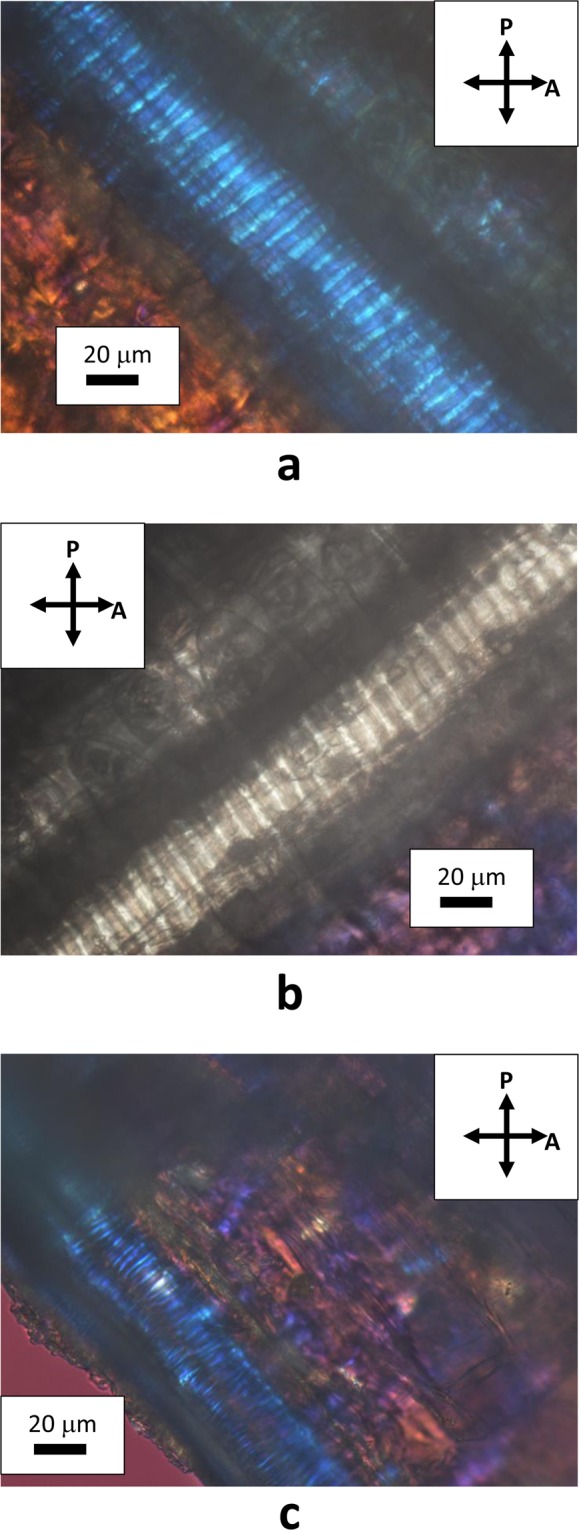
(**a**,**b**) Uncovered fibers viewed through a λ plate and crossed polarizers. The interference color (blue or yellow) changes with the orientation of the fibers from −45° to +45°, as explained in the text, according to references 8–10. Objective Achroplan 40X/0.65, scale length 20 μm (**c**) A fiber seen under circularly polarized light, obtained by using a λ/4 plate between the polarizer and the specimen; artificial colors have been obtained with an additional λ plate. Objective Achroplan 40X/0.65, scale length 20 μm.

**Figure 5:**
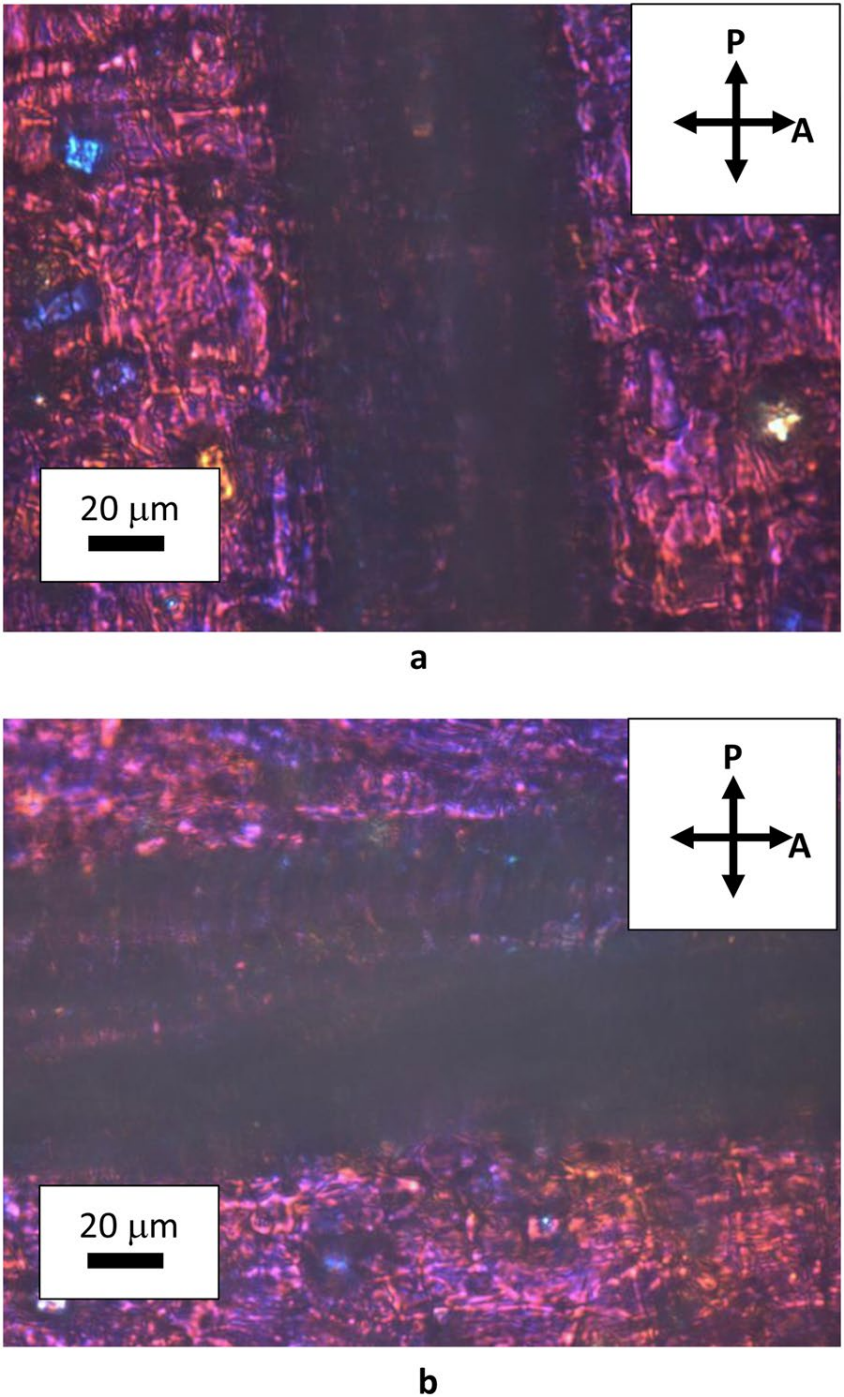
(**a**,**b**) Extinctions of a cellulose fiber observed under crossed polarizers in the presence of a λ plate. Objective Achroplan 40X/0.65, scale length 20 μm. (**a**) Fiber parallel to the polarizer; (**b**) fiber parallel to the analyzer.

**Figure 6:**
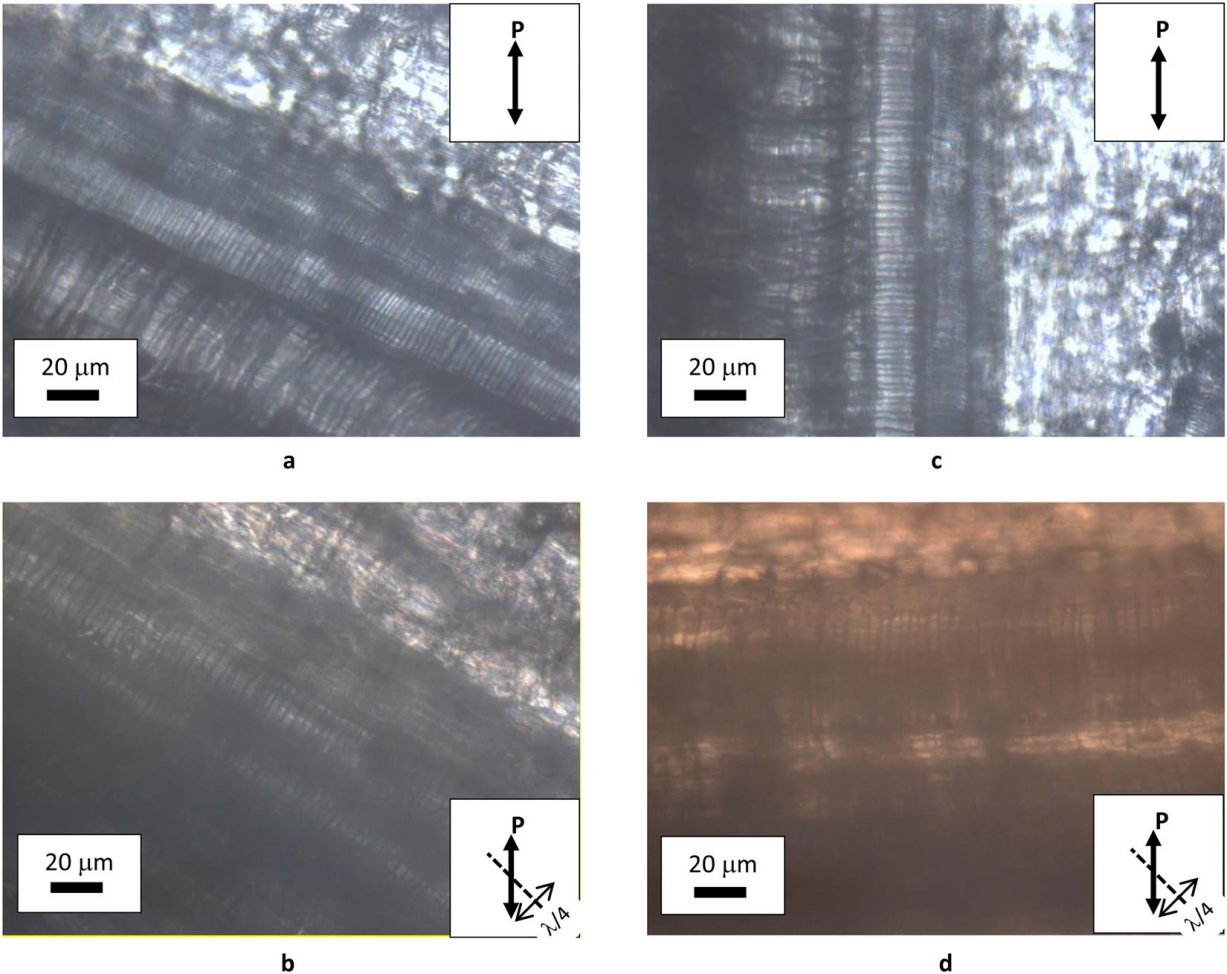
Optical micrographs of cellulose fibers observed without the analyzer, under linearly (**a**,**c**) or circularly (**b**,**d**) incident light. Objective Achroplan 40X/0.65, scale length 20 μm.

Natural cellulose fibers originate from the thickening of walls of vegetal cells that grow in one direction, whereas bi-dimensional growth produces the surrounding matrix of the tissue. Figure 2 shows the assemblage of rounded cells in garlic skin, viewed through crossed polarizes with the addition of a λ plate placed between the objective and the camera. Beneath layers of more or less rounded cells, extended fibers (up to mm in length) formed by one dimensional growth of cells are perceptible as cylindrical protuberances. These fibers become detectable by small adjustments of the focus within the depth of field of an optical microscope. Figure 3 shows one of such structural fibers, covered by flat cells. Occasionally, it is also possible to observe the surface of exposed fibers, as those shown in Fig. 4. Uncovered walls of cellulose fibers show weak birefringence (first order grey), indistinct banding and parallel extinction (Fig. 5). Banding, that is alternate bright and dark strips transversal to the fibers so that two sets of bands with different level of brightness are distinguishable, is present also under circular polarized light and in absence of the analyzer (Fig. 6), appearing even more evident when first order colors are produced, as in Fig. 4, with a λ wave plate. When a birefringent fiber is placed at 45° with respect to the polarization directions, a *nπ*/2 (*n* = 1, 2, 3…) rotation of the stage will produce an equivalent rotation of the ellipsoid axes of the optical indicatrix. Therefore, as already shown in Fig. 1, by observing the fiber with a λ plate also placed at 45° with respect to the polarization directions, the fiber has to change its artificial color from the addition to the subtraction color (or, *vice versa*, from subtraction to addition) for each subsequent *π*/2 rotation of the microscope stage. As shown in Fig. 4, cellulose fibers of garlic skin exhibit yellow (a subtraction color) bands in the first and third quadrants of the stage, and blue (an addition color) bands in the second and fourth quadrants. This pattern conforms to a negative birefringence sign, typical of length-fast fibers. It is worth highlighting that the modulation of birefringence is not due to pressure since it is observable also in absence of object- glasses.

**Figure 7:**
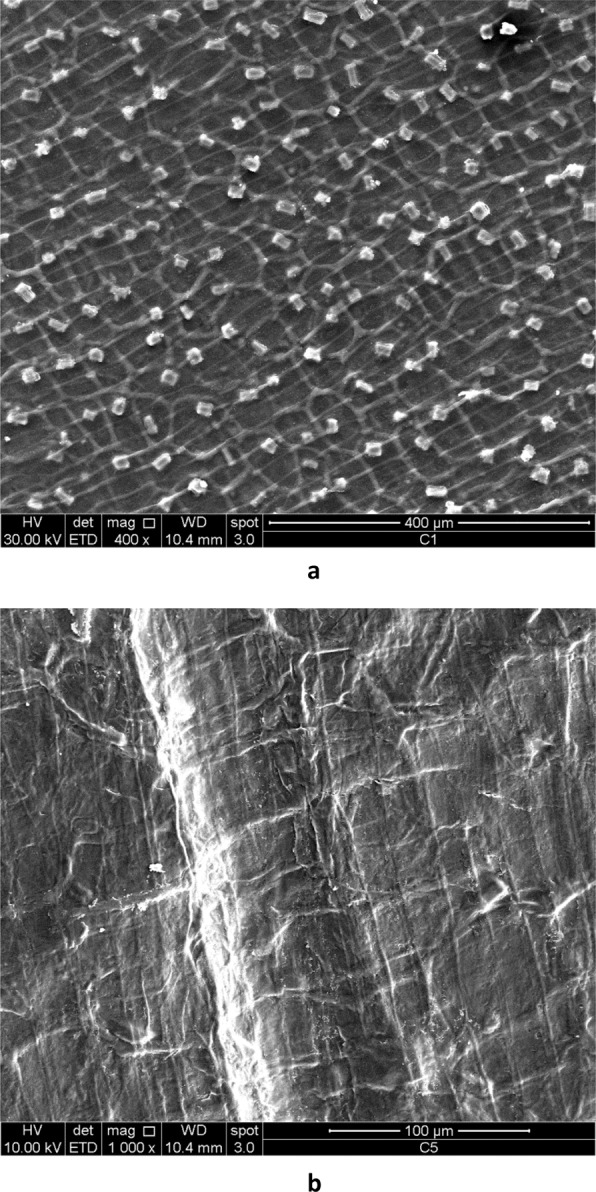
SEM micrographs of metallized surface of garlic skin. A cilindrical fiber is visible in (**b**). Magnification 400X (**a**) and 1000X (**b**).

**Figure 8:**
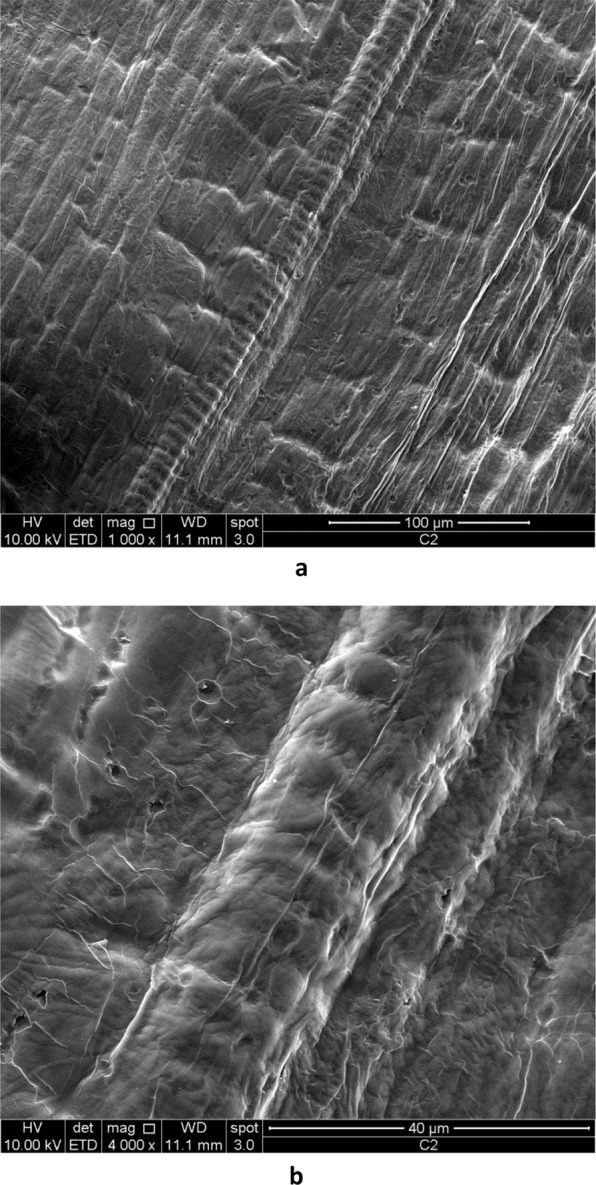
SEM micrographs showing a discontinuous growth of cellulosic fibers. Magnification 1000X (**a**) and 4000X (**b**).

Three-dimensional reproduction of the surface texture of garlic skin has been obtained by SEM, which allows higher depth of focus and magnification than those of an optical microscope, although the high sensitivity to heat of biological tissues, such as garlic skin, does not allow the achievement of very high magnifications without sample damage. The topography of garlic skin emerging from SEM analysis is consistent with the OPM pattern. Indeed, it is evident the similarity between the optical micrographs shown in Figs. 2a, 3 and 4 and the corresponding SEM micrographs shown in Figs. 7a,b and 8. The fibers in Fig. 8 show a transversal banding which reflects a discontinuous growth. The distance between consecutive bands observed by SEM ranges from a few micrometers to a tenth of micrometers, equaling the wideness of bands observed in optical micrographs.

## Discussion

Negative birefringence of an elongated crystal entails that the longitudinal refractive index is lower than the transversal refractive index (length-fast fibers). Assuming a monoclinic unit cell^19^, natural cellulose must be biaxial and should show prevalently an inclined extinction. However, showing parallel extinction, cellulose fibers of garlic skin behave as uniaxial fibers with the optical axis coinciding to the geometrical axis. Therefore, extinction must be caused by the parallel orientation of the constituent crystalline nanofibers^11^ rather than by the crystal structure itself. Moreover, if nanofibrils were not parallel but helicoidally arranged, bands of alternate yellow and blue colors, or a unique greenish color, through a λ plate should be found^8^, because of the different inclination of the nanofibrils in the front and rear walls of the cellulose fiber.

Parallel extinction alone does not allow to discriminate between orientations of nanofibrils parallel and transversal to the fiber axis. However, since cellulose fibers with longitudinal orientation of nanofibrils are positive^11^, the negative birefringence sign found in garlic skin indicates that fibrils are prevalently transversal to the fiber axis.

There are two main explanations of the appearance, under an OPM, of transversal banding along a fiber: interference phenomena, or composition variations. These latter produce also total reflection, that reinforces the brightness of only a set of alternating bands, because of the presence of internal surfaces separating sections with different refractive indices^20^. Let us consider the delay M, measured in wave length, between two coherent beams with the same frequency. If M is an odd multiple of 1/2, the two beams are in opposition and the intensity of light is minimum. If instead M is an even multiple of 1/2, the two beams reinforce each other and the intensity of light is maximum. The expression of the intensity of interference generated by two waves with wideness *h* and *k*, and retardation *δ* measured by phase, is: ${{h}}^{2}+{{k}}^{2}+2{hk}\,\cos \,{\delta }$, thus it varies between ${(h-k)}^{2}$ and ${(h+k)}^{2}$^21^. Therefore, two interfering rays can give the same intensity in two distinct points only by increasing or decreasing M of 1 or its multiples. Moreover, if the delay between the rays is low, the difference in intensity of the constructive bands, relatively to the intensity of each beam, may be too small to be appreciable. The phase difference between the ordinary and extraordinary ray, hence the visibility of the interference fringes in the presence of an analyzer, depends on both the thickness and the orientation of the specimen (i.e. on the difference between the refractive indices associated to the two refracted rays). Let us consider a plate of a uniaxial crystal with thickness *nλ*, where *λ* is some wavelength, and a wavelength *λ*′ close to *λ* so that it is necessary a high value of *n* to satisfy the equality: ${n}({\lambda }{{\prime} }-{\lambda })=(1/2){\lambda }{{\prime} }$. From the latter formula, we have: $n{\lambda }=(n-1/2){\lambda }{{\prime} }$, that is when the interference is constructive for *λ* is destructive for *λ*′ and *vice versa*. However, if *λ*′ and *λ* are very close, their colors are indistinguishable. Let us consider now *λ*′ and *λ* as the wavelengths of the two rays transmitted by an analyzer after the incidence on it of the rays emerging by the anisotropic crystal. Since we have supposed *λ*′ and *λ* very close, also the interference colors produced by the ordinary and extraordinary rays in the analyzer results indistinguishable; interference exist, but it cannot be recognized. However, if the crystal plate is thin, *n* cannot be a very large number and therefore the equality ${n}({\lambda }{{\prime} }-{\lambda })=(1/2){\lambda }{{\prime} }$ can be satisfied only by a wavelength *λ*′ distinguishable by *λ*. In the latter case the interference fringes produced by a thin birefringent crystal in a polarizing microscope, when white light is used, will be visible, since different colors occupies different angular positions within the field of vision.

**Figure 9:**
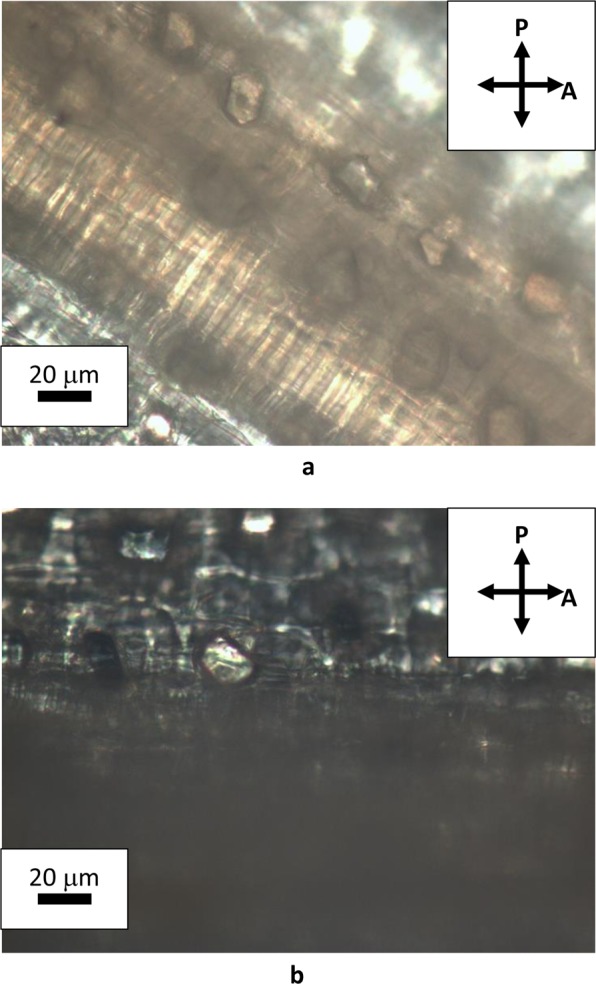
Optical Micrographs of garlic skin. (**a**) Banding is observable under crossed polarizers also with the insertion of a λ/4 plate between the garlic skin and the analyzer, unless the fiber axis is parallel to one of the polarizers, as shown in (**b**). Objective Achroplan 40X/0.65, scale length 20 μm.

**Figure 10:**
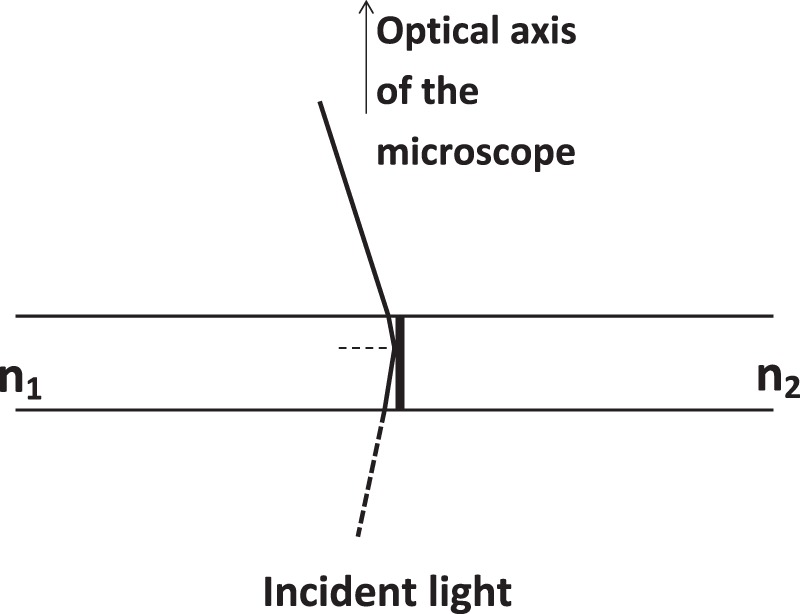
Total reflection upon a vertical interface dividing two means with refractive index n_1_ and n_2_, respectively (n_1_ > n_2_). For the sake of the simplicity, only a refracted ray travelling within the right sector, which is subsequently totally reflected, is shown. All other refracted rays emerging from the top surface of the heterogeneous system, included those coming from the medium n_2_ and propagating within the medium n_1_, are not shown. The reflected ray causes a higher light intensity on the side of the mean with the higher refractive index n_1_. Indeed, total reflection is not possible for light travelling from a medium with the lower refractive index toward a medium with the higher refractive index.

Brightness variations may be due to different causes, such as alternation along the fiber of sections with different composition and, thus, with different optical density^20^ or partial destruction of the parallel orientation of cellulose nanofibrils during fiber growth. To establish the origin of bands, observations have been made with only one polarizer. Indeed, rays with orthogonal polarization cannot interfere before or in absence of the analyzer. Since the analyzer transmits only the components of the incident rays along a certain direction, it changes orthogonal polarization in parallel polarization making possible interference between double refracted rays. By comparing Figs. 5 and 6, it emerges that cellulose fibers oriented parallel to one of the crossed polarizers result dark, whereas if the analyzer is removed feeble but appreciable bands are observable under both linearly and circularly polarized light, whatever the direction of the fiber be. Since under nominal orthoscopic conditions and without an analyzer cellulose fibers appear also banded, bands cannot be exclusively due to interference, in presence of a crossed analyzer, between double refracted rays. Hence, visible bands in cellulose of garlic skin must be ascribed to a difference in brightness caused by fiber heterogeneity. Furthermore, Fig. 9 shows that if between the specimen and the analyzer is placed a λ/4 plate, banding as well as parallel extinction are still observable. With crossed polarizers, interference figures appear as intermittent dark and bright regions typically when conoscopic observations are carried out, since oblique light intercepts differently oriented sections of a material producing different birefringence, optical path and delay values. If the current bands were due to only imperfect orthoscopic conditions, two adjacent bands should not be separated by the Becke line, which is indicative of an abrupt change of the mean refractive index. Colors should instead gradually change from whitish to grey, without a clear separation between them. Furthermore, interference bands of such a kind possess a typical regularity that does not conform to the bands observed in garlic skin. All these findings exclude the visibility of banding patterns due exclusively to refraction of non-orthogonal light with respect to the surfaces of the specimen, indicating a succession along a fiber of pieces with intermittent chemical composition (or physical arrangement) and birefringence power, but with the same optical characteristic as parallel extinction and birefringence sign. Indeed, under real, and hence imperfect orthoscopic observations, light travelling through the fiber from the mean with the higher refractive index to that with the lower refractive index, hits the transversal interface with an angle very close to 90°. As shown in Fig. 10, the incidence angle of the light on a vertical interface within the fiber is hence higher than the limit angle (the angle for which the refracted ray is parallel to the interface), so that the side of the interphase with the higher refractive index will appear brighter because of total reflection. For incident non-polarized light, totally reflected rays are completely polarized only at the Brewster angle, therefore generally they should not give extinction through an analyzer. However, if the incident light is linearly polarized, also the totally reflected light is linearly polarized and its vibration plane is parallel to that of the incident polarized light. Since light in cellulose fibers is divided in two double refracted beams, there will be also two totally reflected rays with orthogonal polarization for each oblique ray incident on the vertical interface, as shown in Fig. 10 (for simplicity only one of the two reflected rays is shown). Under crossed polarizers, hence, totally reflected light undergoes extinction four times by a 2π rotation of the stage, so that the whole fiber undergoes parallel extinction, whereas bands are again visible, by removing the analyzer, also in the directions parallel or normal to the polarizer. The elliptical polarization produced by both the reflected (only due to imperfect orthoscopic conditions) and refracted rays emerging from the top surface of the fiber explains also why the cellulose fibers of garlic show parallel extinction under crossed polarizers and why the extinction remains in the presence of a λ/4 plate between the specimen and the analyzer.

## Conclusions

The observations of elongate and banded structures of garlic skin with a polarizing microscope, under different conditions, show that fibers behave as uniaxial crystals and allow correlations between optical properties and cellulose distribution. Findings lead to the conclusion that the difference in birefringence values along the fiber is not the only cause of transversal banding, and that it is likely accompanied by total reflection of polarized light from a compartment with a higher refractive index to an adjacent sector with a lower refractive index. Therefore, it has been inferred that sections along lignocellulosic fibers undergo an intermittent change of composition, that is a periodic slowdown of cellulose growth accompanied by a more intensive development of the minor components. This conforms to the effect of circadian cycles on the structure and growth of vegetal matters. Moreover, negative birefringence has lead to the conclusion that cellulose nanofibrils are arranged transversally to the geometric axis of the fibers. The present optical procedures, allowing to establish properties-morphology correlations, can be successfully extended to cellulosic fibers of other plant species and are valuable for implementations of mechanical and growth modelling of vegetal systems.

## Materials and Methods

An amount of 0.2 kg of garlic (*Allium sativum*) was peeled and 30 small pieces (c.a. 25 cm^2^ overall) of skin were observed in transmission modality under a polarizing microscope (Axioskop, Zeiss) equipped with a videocamera Micropublisher 5.0 RTV. Photos (2560 × 1920, 300 dpi, 24 bit depth) were acquired by Image Pro Plus software. Two photos (Fig. 4a,b) have been improved with the HDR effect on a single image, using the Canon software Digital Photo Professional 4. The HDR effect has not produced changes others than an uniform increase of vividness. The garlic skin (30–60 μm thick) sandwiched between two object-glasses was observed at room temperature with one or two polarizers, using also a full wave or a quarter wave plate. In order to analyze the state of light emerging from the sample, a λ or λ/4 plate was placed between the specimen and the analyzer, at an angle of 45° with respect to the transmission orientations of the polarizer and the analyzer, according to Fig. 1. To generate circular polarized light, the λ/4 plate was instead inserted between the polarizer and the specimen at an angle of 45° with respect to the transmission orientations of the polarizer and the analyzer. Scanning electron microscopy was performed by using a FEI Quanta 200 FEG (Eindhoven, The Nederlands), after metallization under vacuum of the samples with an Au-Pd alloy by means of a Baltech Med 020 sputtering apparatus.

## Data Availability

The article has no data other than digital micrographs, provided with the manuscript.
